# Infectious Morbidity in Pediatric Patients Receiving Neoadjuvant Chemotherapy for Sarcoma

**DOI:** 10.3390/cancers13091990

**Published:** 2021-04-21

**Authors:** Denise Willmer, Stefan K. Zöllner, Frieder Schaumburg, Heribert Jürgens, Thomas Lehrnbecher, Andreas H. Groll

**Affiliations:** 1 Infectious Disease Research Program, Center for Bone Marrow Transplantation, Department of Pediatric Hematology/Oncology, University Children’s Hospital Münster, 48149 Münster, Germany; d_will06@uni-muenster.de (D.W.); stefan.zoellner@uk-essen.de (S.K.Z.); jurgh@ukmuenster.de (H.J.); 2 Department of Pediatric Oncology & Hematology, Pediatrics III, University Hospital of Essen, 45147 Essen, Germany; 3 Institute of Medical Microbiology, University Hospital Münster, 48149 Münster, Germany; frieder.schaumburg@ukmuenster.de; 4 Pediatric Hematology and Oncology, Hospital for Children and Adolescents, Johann Wolfgang Goethe-University, 60590 Frankfurt am Main, Germany; thomas.lehrnbecher@kgu.de

**Keywords:** infections, cancer, children, bacteremia, pneumonia, sarcoma, solid tumor, outcome

## Abstract

**Simple Summary:**

Infections are an important cause of morbidity and mortality in childhood cancer treatment. The aim of our retrospective study was to assess the infectious burden in pediatric sarcoma patients during neoadjuvant chemotherapy administered according to the EWING 2008, CWS SoTiSaR and EURAMOS clinical trial or registry. Our analyses indicate a substantial infectious morbidity in this group of patients, with 58.8% experiencing at least one episode of febrile neutropenia (FN) and 20.6% at least one microbiologically documented infection (MDI). We also identified parameters that impact on the occurrence of FN and MDIs, including treatment protocol, patient age, and mucositis. These findings may contribute to a better risk stratification for prevention and management of FN and infections as well as for maintaining quality of life, cost control, and optimum outcomes of anticancer treatment.

**Abstract:**

The purpose of this retrospective, single-center cohort study was to assess the infectious burden in pediatric sarcoma patients during neoadjuvant chemotherapy. The review included all patients with a new diagnosis of Ewing sarcoma, osteosarcoma or soft tissue sarcoma between September 2009 and December 2018 who were enrolled in the EWING 2008, CWS SoTiSaR and EURAMOS clinical trial or registry. Primary endpoints were the occurrence of febrile neutropenia (FN) and microbiologically documented infection (MDI). Parameters with a potential impact on FN and MDI were also analyzed. A total of 170 sarcoma patients (median age: 13 years, range: 0–21; 96 m/74 f) received 948 chemotherapy courses (median: 6; range: 2–8). Of these patients, 58.8% had ≥1 FN episode and 20.6% ≥ 1 MDI. FN occurred in 272/948 courses (28.7%) with fever of unknown origin (FUO) in 231 courses and 45 MDI and 19 clinically documented infections (CDI) occurring in a total of 57 courses. Patients enrolled in EWING 2008 had significantly more FN (*p* < 0.001), infections (*p* = 0.02) and MDI (*p* = 0.035). No infection-related deaths were observed. Younger age, tumor type and localization, and higher median and maximum mucositis grades were significantly associated with higher numbers of FN (*p* < 0.001), and younger age (*p* = 0.024) and higher median mucositis grade (*p* = 0.017) with MDI. The study shows substantial infectious morbidity in sarcoma patients during neoadjuvant chemotherapy treatment and opportunities to improve prevention and management.

## 1. Introduction

Febrile neutropenia and documented infections are important causes of morbidity in pediatric patients receiving chemotherapy for cancer [1,2], and optimal infectious disease supportive care in this population relies largely on a precise knowledge of their epidemiology and risk factors. Most information about febrile neutropenia and documented infections stems from studies conducted in patients undergoing treatment for hematological malignancies or allogeneic hematopoietic cell transplantation (HSCT) [3,4,5]. Although prior studies have suggested no substantial differences in the course or outcome of febrile neutropenia and infections between leukemia and solid tumor groups [6], very few studies have focused on febrile neutropenia and infections in pediatric patients with solid tumors, in particular sarcomas [5,7,8,9,10,11]. Given current approaches to antibiotic stewardship and risk-based strategies for management of febrile neutropenia, differentiating the risk for febrile neutropenia and infection for separate entities of solid tumors has become more important.

Sarcoma treatment involves the use of intensive neoadjuvant chemotherapy regimens prior to local therapy that result in transient myelosuppression, which is often complicated by fever and infection [12,13,14,15,16]. Although the safety assessment of induction chemotherapy in the treatment of Ewing sarcoma in the EURO-E.W.I.N.G. 99 clinical trial showed a high incidence of infections [12], little is known about the incidence, risk factors, and outcome of febrile neutropenia and infections during the neoadjuvant chemotherapy treatment phase of pediatric sarcomas. In order to contribute to a better understanding of the burden of infections in this patient population, we analyzed prevalence, risk factors, patterns and outcome of febrile neutropenia and documented infections in pediatric patients with Ewing sarcoma, soft tissue sarcoma, and osteosarcoma who received neoadjuvant chemotherapy according to internationally conducted treatment protocols.

## 2. Patients and Methods

### 2.1. Study Design

The study was a retrospective single-center cohort study of children and adolescents enrolled at the Department of Pediatric Hematology and Oncology, University Children’s Hospital of Muenster, in the EWING 2008 clinical trial and registry, EURAMOS 1 clinical trial and EURAMOS-COSS registry, and CWS SoTiSaR registry treatment protocols between September 2009 and December 2018. Patients received neoadjuvant chemotherapy for Ewing sarcoma, osteosarcoma, or soft tissue sarcoma, respectively. The last follow-up was on 31 January 2020.

The Department serves an area of five million people in the Northwest of Germany and provides specialized care for the entire spectrum of pediatric hematological and oncological disorders. Each year, 140 to 160 patients with a new diagnosis of cancer, 20 to 40 patients with recurrent cancer and approximately 35 patients scheduled to undergo allogeneic HSCT are admitted, accounting for approximately 1200 hospital admissions and close to 15,000 outpatient or day clinic contacts [17].

Patients were identified through the registries of the Department’s clinical research office. Inclusion criteria were: (1) medical care at the Department of Pediatric Hematology and Oncology, Muenster, Germany, (2) a diagnosis of either Ewing sarcoma, osteosarcoma, or soft tissue sarcoma, and (3) receipt of neoadjuvant chemotherapy according to either EWING 2008, CWS SoTiSaR or EURAMOS protocols. Patients not treated according to these protocols, diagnosed in Muenster but treated elsewhere or with an incomplete medical record were excluded. Patient demographics and clinical data were retrieved from the medical information system according to the study protocol by a pseudonymized standardized electronic case report form.

The primary endpoints were episodes of febrile neutropenia (FN) and microbiologically documented infections (MDI). Clinically documented infections (CDI), episodes of fever of unknown origin (FUO), unscheduled hospitalizations, treatment delay, admission to intensive care unit (ICU), and survival through January 2020 were further endpoints. Parameters with a potential impact on FN and MDI were also analyzed. Analyses were performed both on the basis of patients and on the basis of chemotherapy courses, as appropriate.

Written informed consent for data collection and analysis was obtained within the consent procedure for cancer treatment. The study was conducted in accordance with the Declaration of Helsinki, and the protocol was reviewed and approved by the joint Ethics Committee of the University of Muenster and the Chamber of Physicians Westfalen-Lippe (Az 2017-728-f-S; 5 January 2018).

### 2.2. Neoadjuvant Chemotherapy Regimens

Detailed information on the neoadjuvant chemotherapy regimens can be found in the respective protocols [18,19,20]. In brief, the EWING 2008 clinical trial and registry (EWING 2008) protocol stipulated a total of six neoadjuvant courses of vincristine, ifosfamide, doxorubicine, etoposide (VIDE) administered in 21-day intervals depending on hematological recovery. Granulocyte colony-stimulating factor (G-CSF) was recommended, especially when the previous course was complicated by FN, but not mandatory, and MESNA was administered for prevention of urothelial toxicity. G-CSF-supported stem cell mobilization and harvesting was planned after the third course, if indicated. Local therapy was recommended to be performed 21 days after course six, depending on hematological recovery and clinical status [18].

Patients with soft tissue sarcoma enrolled in the CWS SoTiSaR registry (CWS SoTiSaR) were treated according to CWS-Guidance, which recommends courses of different combinations and dosages of actinomycin D (A), adriamycin (Ad), vincristine (V), ifosfamide (I), carboplatinum (C) and etoposide (E) according to risk stratification. G-CSF could be considered, but use was at the discretion of the attending physician. Local therapy was recommended after four courses of neoadjuvant chemotherapy in the majority of risk groups, but more courses were possible [20].

The EURAMOS 1 clinical trial and EURAMOS-COSS registry (EURAMOS) for osteosarcoma patients stipulated a neoadjuvant regimen of MAP, consisting of two cycles each of adriamycin/cisplatinum (AP) in week one plus two separate doses of high-dose methotrexate (M) in weeks 4 and 5, respectively. MAP is sometimes considered as one course, but as chemotherapy feasibility was assessed before each administration of M or AP, we considered each M and each AP as a separate course. G-CSF was recommended when a previous AP cycle had been complicated by FN or prolonged hospitalization (>7 days). Local therapy was planned after the completion of 2 MAP cycles. If needed for logistical reasons, up to two more doses of M were allowed prior to local therapy [19].

### 2.3. Standard of Care

Outpatients presenting with FN or signs of infection were generally hospitalized for diagnostics, treatment, and clinical observation. Patients with FN were evaluated by vital signs, physical examination, interim history, full blood count, c-reactive protein (CRP), liver and renal function, electrolytes, blood and urine cultures prior to the start of antimicrobial therapy. Further diagnostic, e.g., imaging or additional microbiological or biochemical diagnostics, was guided by clinical and laboratory findings.

Initial empirical antibacterial therapy for FN consisted of ceftazidime plus gentamycin until 2016 and was then replaced by piperacillin/tazobactam. Unstable patients were started with meropenem plus vancomycin and were subsequently de-escalated when feasible, based on microbiology laboratory reports. This regimen was also used for escalation in patients with fever persisting for more than 48–72 h or a new fever after defervescence, with or without additional empirical antifungal therapy at the discretion of the attending physician. Patients older than two years of age in stable condition with an anticipated duration of neutropenia of <7 days could be treated with ceftriaxone with the option of outpatient treatment after definite defervescence. Empirical antibacterial therapy was continued until defervescence for at least 48 h and recovery of the neutrophil count (ANC) above 500/µL. Suspected or proven infections were treated according to current management recommendations [21,22].

As standard of care, patients received a surgically implanted central venous catheter prior to the initiation of neoadjuvant chemotherapy. All patients received trimethoprim/sulfamethoxazole 8 mg/msqu twice weekly as prophylaxis for prevention of *Pneumocystis jirovecii* pneumonia, and topical polyenes or azoles for prevention of oropharyngeal candidiasis.

### 2.4. Definitions

Neutropenia was defined as an ANC of <500/µL or assumed when the white blood cell count (WBC) was <1000/µL. Fever was defined as a single oral temperature ≥38.3 °C or temperatures ≥38.0 °C during an 1-h-period [23]. FN was defined as every distinct episode of fever that occurred during neutropenia. FUO was defined as the presence of fever without a clinically or microbiologically identified focus. Organ infection was defined according to the International Pediatric Sepsis Consensus Conference in 2005 [24]. The diagnosis of a blood stream infection (BSI) was based on the detection of the organism in ≥1 blood culture bottle. Invasive fungal disease (IFD) was defined according to the revised definitions of invasive fungal disease by the EORTC/MSGERC consensus group 2020 [25]. Mucositis was defined according to the Common Toxicity Criteria (CTC) for adverse events version 4.03 [26]. Unscheduled hospitalization was defined as any admission to hospital not planned by the treatment protocol. Treatment delay was defined as difference in days between planned and the actual start of the next chemotherapy course. Underweight was defined as a body weight <10th percentile and overweight as a body weight >90th percentile, according to the age- and gender-adjusted German KiGGS reference percentiles [27].

### 2.5. Statistical Analysis

Statistical analysis was performed using Microsoft Excel and IBM SPSS Statistics (version 26, IBM, Armonk, New York, NY, USA) software. Categorial variables were analyzed by chi-square test or Fisher’s exact test, where applicable. Metric variables were analyzed by Mann–Whitney U and Kruskal–Wallis test as appropriate. Parameters with potential impact on FN and MDI were analyzed by bivariate analysis; due to the small sample size and strong correlations between the respective factors in explorative analysis, multivariate analyses were not performed. Similar to the study of Haupt et al. [7], we calculated infection incidence rates for MDI per 100 person-months at risk. Overall and event-free survival were analyzed by Kaplan–Meier curves. All p-values are two-tailed and considered statistically significant at <0.05.

## 3. Results

A total of 195 patients were identified in the study registries, of whom 170 were included in the final analysis. Of the 25 patients excluded, 10 patients were excluded due to the administration of medical treatment at their referring hospitals, nine due to missing indication for chemotherapy, four due to individual treatment concepts and two due to an incomplete medical record.

In a further nine patients, a number of the neoadjuvant chemotherapy courses (14 in total) were not analyzed due to intermittent treatment at hospitals close to home.

### 3.1. Patient Demographics

The demographic and clinical characteristics of the 170 patients are shown in Table 1. Of the 170 patients, 58 (34.1%) were treated in EWING 2008, 50 (29.4%) in the CWS SoTiSaR and 62 (36.5%) in the EURAMOS trial or registry. The median age at diagnosis was 13 years (range 0–21); 96 (56.5%) of the patients were male and 74 (43.5%) were female, and the majority had a body mass index (BMI) between the 10th and the 90th percentile at baseline. Most tumors (62.4%) were localized in the extremities, and approximately one third (37.3%) of patients had metastases at diagnosis. None of the patients had abnormal granulocyte counts at presentation, and, while the case report form did not capture steroid use, only a very few patients may have received glucocorticosteroids to reduce tumor-associated edema. During neoadjuvant chemotherapy, a total of 948 chemotherapy courses were administered (median per patient: 6, range: 2–8) (Table 1).

### 3.2. Overall Infectious and Non-Infectious Patient Morbidity

Table 2 provides an overview of FN episodes, MDIs, CDIs, ICU-admission, weight loss, treatment delay and survival throughout neoadjuvant chemotherapy until local therapy (i.e., surgery or start of radiotherapy). The majority of the patients (*n* = 100; 58.8%) experienced at least one FN episode, and 63 (37.1%) experienced two or more. Comparison across the three different treatment protocols revealed significant differences in both occurrence and frequency of FN episodes with the highest FN morbidity in patients enrolled in EWING 2008, followed by those enrolled in CWS SoTiSaR and those enrolled in EURAMOS (*p* < 0.001). A similar trend across treatment protocols was observed for MDIs that occurred in a total of 20.6% of patients. Of note, there was no apparent difference in MDIs and CDIs between metastatic and non-metastatic disease. Median loss of weight during neoadjuvant chemotherapy was 7.0% of the baseline weight and the median treatment delay accounted for seven days with wide variability but no significant differences among treatment protocols for both parameters. ICU admission occurred in five patients, and all patients survived through local therapy (Table 2).

### 3.3. Febrile Neutropenia and Infections during Chemotherapy

The episodes of febrile neutropenia and infections in the 948 chemotherapy courses are presented in Table 3. Neutropenia was observed in 519 (54.9%) of the 948 chemotherapy courses. Febrile neutropenia occurred in 272 (28.7%) courses with FUO as the final diagnosis in 231 and MDIs or CDIs in 41 courses. In an additional 16 courses, infection was diagnosed in absence of fever, and in 12 of these, the patient was non-neutropenic. In total, 64 documented infections occurred in 57 of the 948 courses (6.0%), of which 45 were microbiologically and 19 clinically documented. Significant differences in frequency between the different treatment protocols were observed for neutropenia (*p* < 0.001), febrile neutropenia (*p* < 0.001), FUO (*p* < 0.001), documented infections (*p* = 0.02) and MDI (*p* = 0.035) with the highest rates in EWING 2008, followed by CWS SoTiSaR and EURAMOS (Table 3).

Among the MDIs, there were 21 BSI, one case of disseminated invasive aspergillosis and 23 infections at various body sites. More than one pathogen was isolated in two BSI episodes. Seventeen of the 23 isolates were Gram-positive organisms with coagulase-negative staphylococci (CoNS) as predominant isolate, and six were Gram-negative rods including two cases of extended-spectrum ß-lactamase (ESBL) producing *Klebsiella* ssp (Table 4). The most common sites for the 23 MDI organ infections were the abdomen, followed by the urogenital system and bone and soft tissues for bacterial infections and the upper respiratory tract followed by the otorhinolaryngeal system for viral infections. Among the 19 CDI organ infections, the upper respiratory tract, the abdomen and bone and soft tissues were the predominantly affected sites (Table S1).

The overall infection rate for MDIs was 7.2 per 100 person-months at risk; the rates for patients treated for Ewing sarcoma, soft tissue sarcoma and osteosarcoma were 9.5, 7.0, and 4.6, respectively. The overall rate for BSI/IFD was 3.5 per 100 person-month at risk and 6.6, 2.7 and 0.5 for the respective entities. Of note, no infection-related deaths occurred during neoadjuvant chemotherapy.

### 3.4. Hospitalization, ICU Admission and Mucositis

Unscheduled hospitalizations for treatment-related adverse events occurred in 37.4% (354/948) of all courses with a median duration of seven days and were significantly more often in EWING 2008 than in CWS SoTiSaR than in EURAMOS (*p* < 0.001). In five courses (0.5%), patients were admitted to the ICU for a median duration of nine days. Admission was for infectious causes in two cases only. As marker of cytotoxicity, CTC grade 1–2 mucositis was recorded in 149 (15.7%) and CTC grade 3–4 in 137 (14.5%) courses. There were significant differences between treatment protocols with higher rates of CTC grade 3–4 mucositis in EWING 2008 than in EURAMOS than in CWS SoTiSaR (Table 3).

### 3.5. Factors Associated with FN and MDI

Younger age was significantly associated with higher number of FN episodes (weak association, Tau *b* = −0.208; *p* < 0.001). Higher number of FN episodes was also significantly associated with longer duration of unscheduled hospitalization (strong association, Tau *b* = 0.665, *p* < 0.001). Significant associations were also found for treatment regimen (*p* < 0.001; EWING 2008 > CWS SoTiSaR > EURAMOS), tumor localization (*p* < 0.001; trunk > extremities), and maximal and median mucositis grade (*p* < 0.001; mucositis grade 3–4 > grade 1–2 > no mucositis) during treatment. No associations were found for sex, metastases, weight at diagnosis, overall treatment delay and weight loss during treatment. Due to its predominant use in patients enrolled in EWING 2008, the effects of G-CSF were not included in the bivariate analyses (Table S2).

Significant associations with the occurrence of MDI by bivariate analysis were found for younger age (*p* = 0.024) and maximal and median mucositis grade (*p* = 0.003 and *p* = 0.017, respectively) with CTC grade 3–4 > CTC grade 1–2 > no mucositis. Presence of MDI was also significantly associated with longer treatment delay (*p* = 0.006) and longer duration of unscheduled hospitalization (*p* = 0.001). No association was found for sex, treatment regimen, tumor localization, metastases, weight at diagnosis and weight loss during therapy (Table S3).

Due to methodological limitations (small sample size and strong correlations between the respective factors in explorative analysis), we decided to abstain from performing multivariate analyses to further substantiate the statistical associations obtained by bivariate analyses.

### 3.6. Overall and Event-Free Survival

After a median duration of follow-up of 4.23 years (range 0.3–10.5), overall cumulative survival at last follow-up one year after the inclusion of the last patient was 68.8% (63.4%, 71.3%, and 70.6% for patients receiving treatment on EWING 2008, CWS SoTiSaR, and EURAMOS, respectively).

The cumulative event-free survival at last follow-up was 59.2% (55.8%, 59.4%, and 62.1% for patients receiving treatment on EWING 2008, CWS SoTiSaR, and EURAMOS, respectively).

## 4. Discussion

Apart from a recent publication on infectious complications in children with malignant bone tumors [11], detailed information on prevalence, risk factors, and outcome of febrile neutropenia and infections in pediatric patients undergoing chemotherapy for sarcoma is scarce. The results presented here document substantial infectious morbidity during the neoadjuvant chemotherapy treatment phase. The majority of the 170 patients (58.8%) experienced at least one FN episode, and 20.6% had at least one MDI. Febrile neutropenia occurred in 272 (28.7%) of the 948 treatment courses, and in 57 (6.0%) courses, a total of 45 microbiologically and 19 clinically documented infections were recorded.

The overall prevalence of FN in our cohort (58.8%) corresponds to data reported by others for pediatric solid tumors [9,28,29]., Patients enrolled in EWING 2008 had a significantly higher prevalence of FN (84.5%) than those enrolled in CWS SoTiSaR and EURAMOS, respectively (Table 3). This rate is also higher than that previously reported in unselected patients with solid tumors [8]. While the relative distribution of FUO and MDI or CDI is overall comparable to that reported in the literature [6,30,31], there was a significantly higher proportion of documented infections in patients enrolled in EWING 2008 relative to patients enrolled in CWS SoTiSaR and EURAMOS.

We observed an overall incidence rate of 7.2 MDI per 100 person-months at risk and of 3.5 BSI/IFD per 100 person-months at risk. These rates are similar to those reported by Haupt et al. who noted an infection rate of 3.2 per 100 person-months at risk for BSI/IFD in their cohort of solid tumors and a rate of 3.4 per 100 person-months at risk for sarcoma patients [7]; these are also similar to data presented by Calton et al. who reported 3.96 BSI per 100 person-months at risk for solid tumors [10]. However, when analyzed by tumor type, we found numerically higher infection rates for patients with Ewing sarcoma (9.5 MDI and 6,6 BSI/IFD per 100 person-months at risk) and lower rates for osteosarcoma patients (4.6 MDI and 0.5 BSI/IFD per 100 person-months at risk), while rates for soft tissue sarcoma patients were similar to the overall infection rate (7.0 MDI and 2,7 BSI/IFD per 100 person-months at risk). A recent retrospective multicenter survey also showed a high risk for infectious complications in patients treated for malignant bone and soft tissue tumors, particular for patients with Ewing sarcoma, during neoadjuvant and adjuvant chemotherapy with higher cumulative incidence rates as in patients with acute lymphoblastic leukemia, non-Hodgkin’s lymphoma and Hodgkin’s disease, and comparable incidence rates to those in patients with acute myeloid leukemia [11]. A study conducted at our center in patients undergoing autologous hematopoietic cell rescue for solid tumors and lymphoma showed an occurrence of non-fungal MDI in 16.5% of the patients [32]. In the analysis presented here, MDI occurred in 20.6% of the patients overall and in 29.3%, 20.0% and 12.9% of patients enrolled in EWING 2008, CWS SoTiSaR and EURAMOS, respectively. Compared to patients undergoing dose intensive chemotherapy with autologous hematopoietic stem cell rescue, who are generally considered to be at high risk of infection, the data from the present study show a comparable, and, in the case of Ewing sarcoma patients, an even higher risk of MDI. However, our findings stand in contrast to the results of a meta-analysis of global individual participant data regarding the prediction of MDI for FN episodes. By univariate analysis, a diagnosis of Ewing sarcoma and osteosarcoma was associated with a decreased risk of MDI [33].

The differences in FN and MDI between the different sarcoma types and treatments and also, tumor localization, may be explained by the differential dose-intensity and mucosal toxicity of the respective regimens [6,30,34], with VIDE combination therapy for Ewing sarcoma being particularly intense [12,14]. Multiagent combination chemotherapy, as used in EWING 2008 and CWS SoTiSaR, may also contribute [35]. An analysis of the potential influence of different chemotherapy courses or individual chemotherapy agents and their cumulative dosages on FN and MDI rates might have provided further associations but was not possible due to methodological limitations. Considering that patients treated for Ewing and soft tissue sarcoma have higher infection rates than the overall population of pediatric solid tumors, and that these rates are comparable to those observed in patients with hematological malignancies, the outcome in our study was favorable, with no infection-related death during neoadjuvant chemotherapy, and only two patients requiring intensive care unit admission for infection-related supportive care.

The spectrum of bacterial species in the 21 episodes of blood stream infection was predominated by CoNS (9/21, Table S1), which is in line with other reports [36]. However, the detection of CoNS raises the question of whether it reflects a true infection or contamination. There is good reason to assume that the detection of CoNS corresponds to true infection as the majority were *S. epidermidis* mainly found in neutropenic patients, both factors that have been associated with an increased likelihood of true infection [37]. Similar to other studies in patients with solid tumors, bone tumors, and solid tumors with autologous hematopoietic stem cell rescue [7,11,32], we observed a low frequency of fungemia and other forms of invasive fungal diseases in sarcoma patients. The only IFD in our cohort was a case of proven pulmonary and disseminated invasive aspergillosis in a patient with Ewing sarcoma following a period of profound granulocytopenia exceeding a duration of twenty days.

Apart from younger age, bivariate analysis identified an association of mucositis with FN and MDI: Higher grades of mucositis correlated with more FN episodes and higher infection rates. These findings are in concordance with the results of other studies that found associations of mucositis with FN [8,38] and severe infections [39] in bivariate analysis. Indeed, mucositis is the result of a complex series of biological events involving inflammation, alteration of the local tissue response, apoptosis of the basal cell layer and loss of mucosal integrity that facilitates migration of mucosal microorganisms and makes the individual prone to invasive bacterial, fungal and viral infection [40,41,42,43,44]. Patients with FN and mucositis are clearly at higher risk of infection, and the presence of mucositis should be considered in approaches to stratify patient management in low and high-risk categories. In addition, strategies to avoid or ameliorate mucositis and the development of treatments that do not cause mucositis should be prioritized in order to reduce infection risks.

Higher numbers of FN and the presence of MDIs were significantly associated with longer duration of unscheduled hospitalization. Longer duration of hospitalization reflects the burden of therapy-induced morbidity and accounts for important use of health care resources. There also was a significant association of MDIs with longer treatment delay, which in turn may contribute to worse event-free survival in the affected patients as has been demonstrated by others for osteosarcoma patients [45,46].

## 5. Conclusions

In conclusion, the data presented here document substantial infectious morbidity in sarcoma patients during the neoadjuvant chemotherapy treatment phase. Prevention and appropriate management of FN and infections are essential to maintain quality of life and cost control, and to avoid treatment delays that may compromise the outcomes of anticancer treatment.

## Figures and Tables

**Table 1:** Demographics and clinical characteristics of 170 patients.

Characteristic	No. (%) or Median (Range)			
	All	EWING 2008	CWS SoTiSaR	EURAMOS
	(*n* = 170)	(*n* = 58)	(*n* = 50)	(*n* = 62)
Age	13 (0–21)	14 (0–21)	9.5 (0–18)	13.5 (2–18)
0–4 years	18 (10.6)	3 (5.2)	13 (26.0)	2 (3.2)
5–9 years	27 (15.9)	9 (15.5)	12 (24.0)	6 (9.7)
10–14 years	72 (42.4)	24 (41.4)	15 (30.0)	33 (53.2)
15–19 years	51 (30.0)	20 (34.5)	10 (20.0)	21 (33.9)
≥20 years	2 (1.2)	2 (3.4)	0	0
Sex				
male	96 (56.5)	36 (62.1)	22 (44.0)	38 (61.3)
female	74 (43.5)	22 (37.9)	28 (56.0)	24 (38.7)
BMI at baseline	18.9 (10.0–38.1)	20.0 (10.0–34.3)	17.6 (11.4–27.0)	19.1 (12.9–38.1)
BMI < P10	26 (15.3)	8 (13.8)	9 (18.0)	9 (14.5)
BMI P10–P90	126 (74.1)	41 (70.7)	38 (76.0)	47 (75.8)
BMI > P90	18 (10.6)	9 (15.5)	3 (6.0)	6 (9.7)
Underlying condition				
Ewing sarcoma	53 (31.2)	53 (91.4)	0	0
osteosarcoma	62 (36.5)	0	0	62 (100)
rhabdomyosarcoma	31 (18.2)	0	31 (62.0)	0
NOS	5 (2.9)	2 (3.4)	3 (6.0)	0
Ewing-like-sarcoma	2 (1.2)	2 (3.4)	0	0
synovial sarcoma	8 (4.7)	0	8 (16.0)	0
PNET	1 (0.6)	1 (1.7)	0	0
infantile fibrosarcoma	1 (0.6)	0	1 (2.0)	0
liposarcoma	1 (0.6)	0	1 (2.0)	0
DSRCT	1 (0.6)	0	1 (2.0)	0
MPNST	2 (1.2)	0	2 (4.0)	0
pleuropulmonary blastoma	2 (1.2)	0	2 (4.0)	0
neuroblastoma ^1^	1 (0.6)	0	1 (2.0)	0
Tumor localization				
extremities	106 (62.4)	30 (51.7)	17 (34.0)	59 (95.2)
trunk	46 (27.1)	24 (41.4)	20 (40.0)	2 (3.2)
other	18 (10.6)	4 (6.9)	13 (26.0)	1 (1.6)
Any metastases at diagnosis	63 (37.3)	29 (50.0)	15 (30.0)	19 (31.1)
distant metastases	56 (33.1)	26 (44.8)	11 (22.0)	19 (31.1)
lung	30 (53.6)	12 (46.2)	3 (27.3)	15 (78.9)
bone	10 (17.9)	6 (23.1)	2 (18.2)	2 (10.5)
lymph nodes	2 (3.6)	0	2 (18.2)	0
other	2 (3.6)	1 (3.8)	1 (9.1)	0
multiple (≥2 organs)	12 (21.4)	7 (26.9)	3 (27.3)	2 (10.5)
regional lymph node infiltration	23 (13.5)	14 (24.1)	8 (16.0)	1 (1.6)
skip lesions	9 (5.3)	6 (10.5)	0	3 (4.8)
Number of chemotherapy courses ^2^	948	341	239	368
per patient	6 (2–8)	6 (3–7)	5 (2–8)	6 (3–8)
G-CSF administration ^3^	67 (39.4)	57 (98.3)	9 (18.0)	1 (1.6)

BMI, Body mass index (kg/m²); P10, 10th perentile and P90, 90th percentile, adapted to age and sex; NOS, not other specified sarcoma; PNET, peripheral neuroectodermal tumour; DSRCT, desmoplastic small round cell tumour; MPNST, malignant peripheral nerve sheath tumour; G-CSF, granulocyte-colony stimulating factor. ^1^ In one patient treated in the CWS protocol diagnosis was changed to neuroblastoma after local therapy. ^2^ VIDE (vincristine, ifosphamide, doxorubicin, etoposide) in 342 (36.1%); M (high-dose methotrexate) in 247 (26.1%), AP (adriamycin, cisplatinum) in 120 (12.7%); I²VA (ifosphamide, vincristine, actinomycin D) in 137 (14.5%) and various other courses of CWS guidance in 102 (10.6%) courses. ^3^ G-CSF was administered in a total of 291 (30.7%) of the courses (41.5% of non-M courses).

**Table 2:** Febrile neutropenia, microbiologically and clinically documented infections, weight loss, treatment delay and survival throughout neoadjuvant chemotherapy until local therapy in 170 patients.

Characteristic	No. (%) or Median (Range)				chi ^2^ Test
	All	EWING 2008	CWS SoTiSaR	EURAMOS	
	(*n* = 170)	(*n* = 58)	(*n* = 50)	(*n* = 62)	*p-*Value
Febrile neutropenia					
≥1 episode of FN	100 (58.8)	49 (84.5)	32 (64.0)	19 (30.6)	<0.001
≥2 episodes of FN	63 (37.1)	40 (69.0)	17 (34.0)	6 (9.7)	<0.001
Microbiologically documented infection					
≥1 episode of MDI	35 (20.6)	17 (29.3)	10 (20.0)	8 (12.9)	0.084
≥2 episodes of MDI	6 (3.5)	4 (6.9)	1 (2.0)	1 (1.6)	0.230
Clinically documented infection					
≥1 episode of CDI	18 (10.6)	8 (13.8)	5 (10.0)	5 (8,1)	0.587
≥2 episodes of CDI	1 (0.6)	1 (1.7)	0	0	0.379
ICU admission	5 (2.9)	2 (3.4)	3 (6.0)	0	0.168
Weight loss during chemotherapy in %	7.0 (0–31.2)	7.2 (0–25.1)	4.1 (0–17.9)	8.4 (0–31.2)	
Overall treatment delay in days	7.0 (0–61)	8.5 (0–61)	4.0 (0–23)	9.0 (0–48)	
Survival through local therapy	165 (100) ^1,2,3^	57 (100) ^1^	49 (100) ^2^	59 (100) ^3^	

FN, febrile neutropenia; MDI, microbiologically documented infection; CDI, clinically documented infection; ICU, intensive care unit. ^1^ 1 missing value, 1 censored due treatment discontinuation. ^2^ 1 missing value. ^3^ 1 censored due to treatment discontinuation, 1 censored due to treatment modification.

**Table 3:** Neutropenia, febrile neutropenia, FUO, documented infections, unscheduled hospitalisation, ICU admission and mucositis in 948 courses.

Characteristic	No. (%) of Courses or Median (Range)				chi ^2^ Test
	All	EWING 2008	CWS SoTiSaR	EURAMOS	*p*-Value
	(*n* = 948)	(*n* = 341)	(*n* = 239)	(*n* = 368)	
Neutropenia	519 (54.9)	321 (94.1)	157 (65.7)	41 (11.2)	<0.001
Febrile neutropenia	272 (28.7)	179 (52.5)	67 (28.0)	26 (7.1)	<0.001
FUO	231 (24.4)	152 (44.6)	55 (23.0)	24 (6.5)	<0.001
Documented infections ^1^	64 (6.0 ^1^)	32 (8.5 ^1^)	18 (6.3 ^1^)	14 (3.5 ^1^)	0.020
MDIs ^1^	45 (4.4 ^1^)	23 (6.5 ^1^)	13 (4.6 ^1^)	9 (2.4 ^1^)	0.035
CDIs	19 (2.0)	9 (2.6)	5 (2.1)	5 (1.4)	0.475
Unscheduled hospitalization	354 (37.4)	192 (56.3)	79 (33.2)	83 (22.6)	<0.001
duration in days	7.0 (1–23)	7.0 (2–21)	5.0 (2–21)	7.0 (1–23)	
ICU admission	5 (0.5)	2 (0.6)	3 (1.3)	0	0.112
duration in days	9.0 (2–21)	5.0 (2–8)	11.0 (8–21)		
infectious cause	2	2	0		
Mucositis					<0.001
no mucositis	662 (69.8)	109 (32.0)	225 (94.1)	328 (89.1)	
CTC grade 1–2	149 (15.7)	138 (40.5)	3 (1.3)	8 (2.2)	
CTC grade 3–4	137 (14.5)	94 (27.6)	11 (4.6)	32 (8.7)	

FUO, fever of unknown origin; MDI, microbiologically documented infection; CDI, clinically documented infection; ICU, intensive care unit; CTC, common toxicity criteria. ^1^ 5 courses with 2 infectious episodes, 1 course with 3 infectious episodes; in 16 courses infections occurred in absence of FN (2 EWING, 3 CWS, 11 EURAMOS); of those in 12 in absence of neutropenia (0 EWING, 1 CWS, 11 EURAMOS), please see text.

**Table 4:** Overview of 45 * MDI infections and 19 CDI infections in 948 chemotherapy courses.

Infection	MDI (No.)			CDI (No.)
	Bacterial	Fungal	Viral	
	Gram+/Gram−			
Blood stream infection	17/6 ^1^	-	-	-
Systemic infection	0/0	1 ^6^	1 ^7^	-
Organ infection	-	-	-	-
Central nervous system	1/0 ^2^	-	-	-
Oropharynx	-	-	3 ^8^	2 ^10^
Upper respiratory tract	-	-	7 ^9^	5 ^11^
Lung	-	-	-	2 ^12^
Abdomen and gastrointestinal tract	7/0 ^3^	-	-	4 ^13^
Urogenital tract	2/2 ^4^	-	-	2 ^14^
Bone and soft tissues	1/3 ^5^	-	-	4 ^15^

MDI, microbiologically documented infection; CDI, clinically documented infection. * 4 infections with multiple pathogens. ^1^ for detail, consult Supplementary Materials, Table S1. ^2^ *Staphylococcus* hominis. ^3^ *Clostridioides difficile* colitis (6), *enteropathogenic Staphylococcus aureus* enteritis (1). ^4^ *Enterococcus faecalis* (2), *Pseudomonas aeruginosa* (1), *Enterobacter cloacae* (1). ^5^ wound infection with *Enterococcus faecalis*, *Pseudomonas luteola* and *Bacteroides sp.* (1), axillary abscess with *Escherichia coli* (1). ^6^ invasive pulmonary aspergillosis with dissemination in liver and spleen (1). ^7^ *primary Epstein-Barr virus infection*. ^8^ *Herpes simplex virus stomatitis* (3). ^9^ *human Metapneumovirus* (1), *Influenza A virus* (1), *Parainfluenza 2 virus* (1), *Rhinovirus* (1), *Enterovirus* (1), *Respiratory syncycial virus* (2). ^10^ tonsillitis (1), dental abscess (1). ^11^ unspecific upper respiratory tract infections (5). ^12^ pneumonia (2; 1 with respiratory insufficiency). ^13^ gastroenteritis (1), colitis (2), migratory peritonitis (1). ^14^ urinary tract infections (2; 1 with transurethral indwelling catheter). ^15^ port catheter site infection (1), panaritium (3); without documented microbacterial cause.

## Data Availability

The data presented in this study are available on request from the corresponding author.

## Supplementary Materials

The following are available online at https://www.mdpi.com/article/10.3390/cancers13091990/s1, Table S1: Overview of 21 blood stream infections in 19 patients. Table S2: Bivariate analysis of potential factors associated to the number of FN episodes during neoadjuvant chemotherapy in 170 patients. Table S3: Bivariate analysis of potential factors associated with development of ≥ MDI episode during neoadjuvant chemotherapy in 170 patients.

## References

[1] Ammann R.A., Tissing W.J.E., Phillips B. (2012). Rationalizing the approach to children with fever in neutropenia. Curr. Opin. Infect. Dis..

[2] Bodey G.P., Buckley M., Sathe Y.S., Freireich E.J. (1966). Quantitative relationships between circulating leukocytes and infection in patients with acute leukemia. Ann. Intern. Med..

[3] Katsimpardi K., Papadakis V., Pangalis A., Parcharidou A., Panagiotou J.P., Soutis M., Papandreou E., Polychronopoulou S., Haidas S. (2006). Infections in a pediatric patient cohort with acute lymphoblastic leukemia during the entire course of treatment. Support. Care Cancer.

[4] Srinivasan A., McLaughlin L., Wang C., Srivastava D.K., Shook D.R., Leung W., Hayden R.T. (2014). Early infections after autologous hematopoietic stem cell transplantation in children and adolescents: The St. Jude experience. Transpl. Infect. Dis..

[5] Choi Y.B., Yi E.S., Kang J.-M., Lee J.W., Yoo K.H., Kim Y.-J., Sung K.W., Koo H.H. (2016). Infectious Complications during Tandem High-Dose Chemotherapy and Autologous Stem Cell Transplantation for Children with High-Risk or Recurrent Solid Tumors. PLoS ONE.

[6] Koçak U., Rolston K.V.I., Mullen C.A. (2002). Fever and neutropenia in children with solid tumors is similar in severity and outcome to that in children with leukemia. Support. Care Cancer.

[7] Haupt R., Romanengo M., Fears T.R., Viscoli C., Castagnola E. (2001). Incidence of septicaemias and invasive mycoses in children undergoing treatment for solid tumours: A 12-year experience at a single Italian institution. Eur. J. Cancer.

[8] Castelán-Martínez O.D., Rodríguez-Islas F., Vargas-Neri J.L., Palomo-Colli M.A., López-Aguilar E., Clark P., Castañeda-Hernádez G., Rivas-Ruiz R. (2016). Risk Factors for Febrile Neutropenia in Children With Solid Tumors Treated With Cisplatin-based Chemotherapy. J. Pediatr. Hematol. Oncol..

[9] Garrido M.M., Garrido R.Q., Cunha T.N., Ehrlich S., Martins I.S. (2019). Comparison of epidemiological, clinical and microbiological characteristics of bloodstream infection in children with solid tumours and haematological malignancies. Epidemiol. Infect..

[10] Calton E.A., Doaré K.L., Appleby G., Chisholm J.C., Sharland M., Ladhani S.N. (2014). Invasive bacterial and fungal infections in paediatric patients with cancer: Incidence, risk factors, aetiology and outcomes in a UK regional cohort 2009–2011. Pediatr. Blood Cancer.

[11] Czyzewski K., Galazka P., Zalas-Wiecek P., Gryniewicz-Kwiatkowska O., Gietka A., Semczuk K., Chelmecka-Wiktorczyk L., Zak I., Salamonowicz M., Fraczkiewicz J. (2019). Infectious complications in children with malignant bone tumors: A multicenter nationwide study. Infect. Drug Resist..

[12] Juergens C., Weston C., Lewis I., Whelan J., Paulussen M., Oberlin O., Michon J., Zoubek A., Juergens H., Craft A. (2006). Safety assessment of intensive induction with vincristine, ifosfamide, doxorubicin, and etoposide (VIDE) in the treatment of Ewing tumors in the EURO-E.W.I.N.G. 99 clinical trial. Pediatr. Blood Cancer.

[13] Whelan J.S., Bielack S.S., Marina N., Smeland S., Jovic G., Hook J.M., Krailo M., Anninga J., Butterfass-Bahloul T., Böhling T. (2015). EURAMOS-1, an international randomised study for osteosarcoma: Results from pre-randomisation treatment. Ann. Oncol..

[14] Strauss S.J., McTiernan A., Driver D., Hall-Craggs M., Sandison A., Cassoni A.M., Kilby A., Michelagnoli M., Pringle J., Cobb J. (2003). Single center experience of a new intensive induction therapy for ewing’s family of tumors: Feasibility, toxicity, and stem cell mobilization properties. J. Clin. Oncol..

[15] Gupta A.A., Anderson J.R., Pappo A.S., Spunt S.L., Dasgupta R., Indelicato D.J., Hawkins D.S. (2012). Patterns of chemotherapy-induced toxicities in younger children and adolescents with rhabdomyosarcoma: A report from the Children’s Oncology Group Soft Tissue Sarcoma Committee. Cancer.

[16] Paulussen M., Fröhlich B., Jürgens H. (2001). Ewing tumour: Incidence, prognosis and treatment options. Paediatr. Drugs.

[17] Universitätsklinikum Münster: Klinik für Kinder-und Jugendmedizin—Pädiatrische Hämatologie und Onkologie Klinik in Zahlen. https://www.ukm.de/index.php?id=4372.

[18] EU Clinical Trials Register EWING 2008: EduraCT Number 2008-003658-13. https://www.clinicaltrialsregister.eu/ctr-search/trial/2008-003658-13/DE.

[19] EU Clinical Trials Register EURAMOS 1: EduraCT Number 2004-000242-20. https://www.clinicaltrialsregister.eu/ctr-search/trial/2004-000242-20/GB.

[20] Cooperative Weichteilsarkom Studiengruppe der GPOH Registry for Soft Tissue Sarcoma and Other Soft Tissue Tumours in Children, Adolescents, and Young Adults: CWS-SoTiSaR. https://www.kinderkrebsinfo.de/health_professionals/clinical_trials/pohkinderkrebsinfotherapiestudien/cws_sotisar/index_eng.html.

[21] Berner R., Bialek R., Forster J., Härtel C., Heininger U., Huppertz H.-I., Liese J.G., Nadal D., Simon A. (2018). DGPI-Handbuch: Infektionen bei Kindern und Jugendlichen.

[22] Deutsche Gesellschaft für Pädiatrische Infektiologie (DGPI), Gesellschaft für Pädiatrische Onkologie und Hämatologie AWMF S2K Leitlinie—Diagnostik und Therapie bei Kindern mit Onkologischer Grunderkrankung, Fieber und Granulozytopenie (mit Febriler Neutropenie) Außerhalb der Allogenen Stammzelltransplantation: AWMF-Registernummer 048/14. https://www.awmf.org/leitlinien/detail/ll/048-014.html.

[23] Freifeld A.G., Bow E.J., Sepkowitz K.A., Boeckh M.J., Ito J.I., Mullen C.A., Raad I.I., Rolston K.V., Young J.-A.H., Wingard J.R. (2011). Clinical practice guideline for the use of antimicrobial agents in neutropenic patients with cancer: 2010 update by the infectious diseases society of america. Clin. Infect. Dis..

[24] Goldstein B., Giroir B., Randolph A. (2005). International pediatric sepsis consensus conference: Definitions for sepsis and organ dysfunction in pediatrics. Pediatr. Crit. Care Med..

[25] Donnelly J.P., Chen S.C., Kauffman C.A., Steinbach W.J., Baddley J.W., Verweij P.E., Clancy C.J., Wingard J.R., Lockhart S.R., Groll A.H. (2020). Revision and Update of the Consensus Definitions of Invasive Fungal Disease From the European Organization for Research and Treatment of Cancer and the Mycoses Study Group Education and Research Consortium. Clin. Infect. Dis..

[26] National Cancer Institute Common Terminology Criteria for Adverse Events (CTCAE). https://ctep.cancer.gov/protocolDevelopment/electronic_applications/ctc.htm#ctc_40.

[27] Robert Koch-Institut—Referenzperzentile für Anthropometrische Maßzahlen und Blutdruck aus der Studie zur Gesundheit von Kindern und Jugendlichen in Deutschland (KiGGS). https://www.rki.de/DE/Content/Gesundheitsmonitoring/Gesundheitsberichterstattung/GBEDownloadsB/KiGGS_Referenzperzentile.html.

[28] Ammann R.A., Aebi C., Hirt A., Ridolfi Lüthy A. (2004). Fever in neutropenia in children and adolescents: Evolution over time of main characteristics in a single center, 1993–2001. Support. Care Cancer.

[29] Alexander S.W., Wade K.C., Hibberd P.L., Parsons S.K. (2002). Evaluation of Risk Prediction Criteria for Episodes of Febrile Neutropenia in Children with Cancer. J. Pediatr. Hematol. Oncol..

[30] Castagnola E., Fontana V., Caviglia I., Caruso S., Faraci M., Fioredda F., Garrè M.L., Moroni C., Conte M., Losurdo G. (2007). A prospective study on the epidemiology of febrile episodes during chemotherapy-induced neutropenia in children with cancer or after hemopoietic stem cell transplantation. Clin. Infect. Dis..

[31] Agyeman P., Aebi C., Hirt A., Niggli F.K., Nadal D., Simon A., Ozsahin H., Kontny U., Kühne T., Beck Popovic M. (2011). Predicting bacteremia in children with cancer and fever in chemotherapy-induced neutropenia: Results of the prospective multicenter SPOG 2003 FN study. Pediatr. Infect. Dis. J..

[32] Linke C., Tragiannidis A., Ahlmann M., Fröhlich B., Wältermann M., Burkhardt B., Rossig C., Groll A.H. (2019). Epidemiology and management burden of invasive fungal infections after autologous hematopoietic stem cell transplantation: 10-year experience at a European Pediatric Cancer Center. Mycoses.

[33] Phillips R.S., Sung L., Ammann R.A., Riley R.D., Castagnola E., Haeusler G.M., Klaassen R., Tissing W.J.E., Lehrnbecher T., Chisholm J. (2016). Predicting microbiologically defined infection in febrile neutropenic episodes in children: Global individual participant data multivariable meta-analysis. Br. J. Cancer.

[34] Bagnasco F., Haupt R., Fontana V., Valsecchi M.G., Rebora P., Caviglia I., Caruso S., Castagnola E. (2012). Risk of repeated febrile episodes during chemotherapy-induced granulocytopenia in children with cancer: A prospective single center study. J. Chemother..

[35] Ouyang Z., Peng D., Dhakal D.P. (2013). Risk factors for hematological toxicity of chemotherapy for bone and soft tissue sarcoma. Oncol. Lett..

[36] Simon A., Furtwängler R., Graf N., Laws H.J., Voigt S., Piening B., Geffers C., Agyeman P., Ammann R.A. (2016). Surveillance of bloodstream infections in pediatric cancer centers—what have we learned and how do we move on?. GMS Hyg. Infect. Control.

[37] García-Vázquez E., Fernández-Rufete A., Hernández-Torres A., Canteras M., Ruiz J., Gómez J. (2013). When is coagulase-negative Staphylococcus bacteraemia clinically significant?. Scand. J. Infect. Dis..

[38] Rondinelli P.I.P., Ribeiro K.d.C.B., de Camargo B. (2006). A proposed score for predicting severe infection complications in children with chemotherapy-induced febrile neutropenia. J. Pediatr. Hematol. Oncol..

[39] Badiei Z., Khalesi M., Alami M.H., Kianifar H.R., Banihashem A., Farhangi H., Razavi A.R. (2011). Risk factors associated with life-threatening infections in children with febrile neutropenia: A data mining approach. J. Pediatr. Hematol. Oncol..

[40] Al-Dasooqi N., Sonis S.T., Bowen J.M., Bateman E., Blijlevens N., Gibson R.J., Logan R.M., Nair R.G., Stringer A.M., Yazbeck R. (2013). Emerging evidence on the pathobiology of mucositis. Support. Care Cancer.

[41] Sobue T., Bertolini M., Thompson A., Peterson D.E., Diaz P.I., Dongari-Bagtzoglou A. (2018). Chemotherapy-induced oral mucositis and associated infections in a novel organotypic model. Mol. Oral Microbiol..

[42] Villa A., Sonis S.T. (2015). Mucositis: Pathobiology and management. Curr. Opin. Oncol..

[43] Hong J., Park H.-K., Park S., Lee A., Lee Y.-H., Shin D.-Y., Koh Y., Choi J.-Y., Yoon S.-S., Choi Y. (2020). Strong association between herpes simplex virus-1 and chemotherapy-induced oral mucositis in patients with hematologic malignancies. Korean J. Intern. Med..

[44] Katagiri H., Fukui K., Nakamura K., Tanaka A. (2018). Systemic hematogenous dissemination of mouse oral candidiasis is induced by oral mucositis. Odontology.

[45] Vasquez L., Silva J., Chavez S., Zapata A., Diaz R., Tarrillo F., Maza I., Sialer L., García J. (2020). Prognostic impact of diagnostic and treatment delays in children with osteosarcoma. Pediatr. Blood Cancer.

[46] Abou Ali B., Salman M., Ghanem K.M., Boulos F., Haidar R., Saghieh S., Akel S., Muwakkit S.A., El-Solh H., Saab R. (2019). Clinical Prognostic Factors and Outcome in Pediatric Osteosarcoma: Effect of Delay in Local Control and Degree of Necrosis in a Multidisciplinary Setting in Lebanon. J. Glob. Oncol..

