# Supplementary material for: Infectious Morbidity in Pediatric Patients Receiving Neoadjuvant Chemotherapy for Sarcoma
Source: Cancers (Basel). 2021 Apr 21;13(9):1990. doi: 10.3390/cancers13091990 (PMC8122626; doi:10.3390/cancers13091990)
Supplement: Supplementary file 1 [file cancers-13-01990-s001.zip › cancers-1156165-supplementary.pdf]

Supplementary Material: Infectious Morbidity in Pediatric Patients Receiving Neoadjuvant Chemotherapy for Sarcoma

Denise Willmer, Stefan K. Zöllner, Frieder Schaumburg, Heribert Jürgens, Thomas Lehrnbecher and Andreas H. Groll

Table S1. Overview of 21 blood stream infections in 19 patients.

		Pathogen	Sex	Age	Diagnosis	Course
Gram positive	CoNS	*Staphylococcus epidermidis*	male	13	Ewing sarcoma	VIDE
		Staphylococcus epidermidis	female[1]	14	Ewing sarcoma	VIDE
		Staphylococcus epidermidis	male	11	Ewing sarcoma	VIDE
		Staphylococcus epidermidis	female	11	Ewing sarcoma	VIDE
		Staphylococcus epidermidis	female	0	Rhabdomyosarcoma	I²VA
		Staphylococcus epidermidis	male	5	Rhabdomyosarcoma	I²VA
		Staphylococcus epidermidis	female	7	Rhabdomyosarcoma	I²VA
		Staphylococcus haemolyticus	male	17	Osteosarcoma	M
	streptococci	*Streptococcus mitis*	male[2]	16	Ewing sarcoma	VIDE
	enterococci	*Enterococcus faecalis*	male	0	PNET	VIDE
		Enterococcus faecalis	female	10	Ewing sarcoma	VIDE
		Enterococcus faecium	male	6	Ewing sarcoma	VIDE
	other	*Micrococcus luteus*	female	14	Ewing sarcoma	VIDE
		Brevibacillus borstelensis	female	18	Ewing sarcoma	VIDE
		Brevibacterium casei	male[2]	16	Ewing sarcoma	VIDE
Gram negative	enterobacterales	*Escherischia coli*	female	12	Ewing sarcoma	VIDE
		Klebsiella pneumoniae (ESBL)	female	18	Rhabdomyosarcoma	I²VAd²
		Enterobacter cloacae complex	female	1	Rhabdomyosarcoma	I²VA
	other	*Moraxella osloensis*	male	11	Ewing sarcoma	VIDE
Mixed		*Micrococcus luteus, Paracoccus yecii*	female[1]	14	Ewing sarcoma	VIDE
		Staphylococcus hominis, Klebsiella oxycota (ESBL)	male	8	Ewing sarcoma	VIDE

CoNS, coagulase negative staphylococci; ESBL, extended-spectrum ß-lactamase; V, vincristine; I, ifosfamid; D, doxorubicine; E, etoposide; A, actinomycin D; M, methotrexate; Ad, adriamycin [1] same patient; [2] same patient.

Table S2. Bivariate analysis of potential factors associated to the number of FN episodes during neoadjuvant chemotherapy in 170 patients.

Characteristic	Median Number of FN Episodes (Range)	*p*-value
Sex		0.127 [a]
male	1.0 (0–6)	
female	1.0 (0–6)	
Any metastases		0.942 [a]
no	1.0 (0–6)	
yes	1.0 (0–6)	
Treatment regimen		< 0.001 [b]
EWING 2008	3.0 (0–6)	
EURAMOS	0.0 (0–3)	
CWS SoTiSaR	1.0 (0–6)	
EWING 2008 vs. EURAMOS		< 0.001 [c]
EWING 2008 vs. CWS SoTiSaR		< 0.001 [c]
EURAMOS vs. CWS SoTiSaR		< 0.001 [c]
Tumour localisation		< 0.001 [b]
extremities	0.0 (0–6)	
trunk	2.5 (0–6)	
other	1.0 (0–6)	
extremities vs. trunk		< 0.001 [c]
extremities vs. other		0.066 [c]
trunk vs. other		0.208 [c]
Maximal mucositis grade		< 0.001 [b]

no mucositis	0.0 (0–6)	
grade 1–2	1.0 (0–6)	
grade 3–4	2.0 (0–6)	
no mucositis vs. grade 1–2		<0.001 [c]
no mucositis vs. grade 3–4		<0.001 [c]
grade 1–2 vs. grade 3–4		0.166 [c]
Median mucositis grade		< 0.001 [b]
no mucositis	0.0 (0–6)	
grade 1–2	2.0 (0–6)	
grade 3–4	5.0 (0–6)	
no mucositis vs. grade 1–2		<0.001 [c]
no mucositis vs. grade 3–4		<0.001 [c]
grade 1–2 vs. grade 3–4		0.014 [c]
Weight at diagnosis		0.264 [b]
normal weight	1.0 (0–6)	
underweight	1.0 (0–6)	
overweight	1.0 (0–6)	
Age in years	−0.208	< 0.001 [d]
Weight loss during therapy	0.121	0.037 [d]
Treatment delay in days	0.140	0.016 [d]
Duration of unscheduled hospitalisation in days	0.665	< 0.001 [d]

FN, febrile neutropenia; ES, Ewing sarcoma; OS, osteosarcoma; STS, soft tissue sarcoma; G-CSF, granulocyte stimulating factor [a] Mann-Whitney U test; [b] Kruskal-Wallis-test; [c] posthoc Mann-Whitney U test; [d] Kendall's Tau-b Please note that G-CSF administration was not analysed due to the predominant use of G-CSF in Ewing sarcoma patients (see Table 1).

Table S3. Bivariate analysis of potential factors associated to the number of FN episodes during neoadjuvant chemotherapy in 170 patients.

**Characteristic**	**Median Number of FN Episodes (Range)**	***p*-value**
Sex		0.127 [a]
male	1.0 (0–6)	
female	1.0 (0–6)	
Any metastases		0.942 [a]
no	1.0 (0–6)	
yes	1.0 (0–6)	
Treatment regimen		< 0.001 [b]
EWING 2008	3.0 (0–6)	
EURAMOS	0.0 (0–3)	
CWS SoTiSaR	1.0 (0–6)	
EWING 2008 vs. EURAMOS		< 0.001 [c]
EWING 2008 vs. CWS SoTiSaR		< 0.001 [c]
EURAMOS vs. CWS SoTiSaR		< 0.001 [c]
Tumour localisation		< 0.001 [b]
extremities	0.0 (0–6)	
trunk	2.5 (0–6)	
other	1.0 (0–6)	
extremities vs. trunk		< 0.001 [c]
extremities vs. other		0.066 [c]
trunk vs. other		0.208 [c]
Maximal mucositis grade		< 0.001 [b]
no mucositis	0.0 (0–6)	
grade 1–2	1.0 (0–6)	
grade 3–4	2.0 (0–6)	
no mucositis vs. grade 1–2		<0.001 [c]
no mucositis vs. grade 3–4		<0.001 [c]
grade 1–2 vs. grade 3–4		0.166 [c]
Median mucositis grade		< 0.001 [b]

no mucositis	0.0 (0–6)	
grade 1–2	2.0 (0–6)	
grade 3–4	5.0 (0–6)	
no mucositis vs. grade 1–2		<0.001 [c]
no mucositis vs. grade 3–4		<0.001 [c]
grade 1–2 vs. grade 3–4		0.014 [c]
Weight at diagnosis		0.264 [b]
normal weight	1.0 (0–6)	
underweight	1.0 (0–6)	
overweight	1.0 (0–6)	
	Kendall's–Tau–b correlation coefficient	*p*–value
Age in years	−0.208	< 0.001 [d]
Weight loss during therapy	0.121	0.037 [d]
Treatment delay in days	0.140	0.016 [d]
Duration of unscheduled hospitalisation in days	0.665	< 0.001 [d]

FN, febrile neutropenia; ES, Ewing sarcoma; OS, osteosarcoma; STS, soft tissue sarcoma; G-CSF, granulocyte stimulating factor [a] Mann-Whitney U test; [b] Kruskal-Wallis-test; [c] posthoc Mann-Whitney U test; [d] Kendall's Tau-b; Please note that G-CSF administration was not analysed due to the predominant use of G-CSF in Ewing sarcoma patients (see Table 1).
